# Credibility, Accuracy, and Comprehensiveness of Internet-Based Information About Low Back Pain: A Systematic Review

**DOI:** 10.2196/13357

**Published:** 2019-05-07

**Authors:** Giovanni Ferreira, Adrian C Traeger, Gustavo Machado, Mary O'Keeffe, Christopher G Maher

**Affiliations:** 1 Institute for Musculoskeletal Health, Sydney School of Public Health, Faculty of Medicine and Health The University of Sydney Sydney Australia

**Keywords:** medical informatics, low back pain, patient portals, systematic review, consumer health information

## Abstract

**Background:**

Low back pain (LBP) affects millions of people worldwide, and misconceptions about effective treatment options for this condition are very common. Websites sponsored by organizations recognized as trustworthy by the public, such as government agencies, hospitals, universities, professional associations, health care organizations and consumer organizations are an important source of health information for many people. However, the content of these websites regarding treatment recommendations for LBP has not been fully evaluated.

**Objective:**

This study aimed to determine the credibility, accuracy, and comprehensiveness of treatment recommendations for LBP in noncommercial, freely accessible websites.

**Methods:**

We conducted a systematic review of websites from government agencies, hospitals, universities, professional associations, health care organizations and consumer organizations. We conducted searches on Google. Treatment recommendations were coded based on the 2016 National Institute for Health and Care Excellence (NICE) guidelines and the 2017 American College of Physicians guideline on LBP. Primary outcomes were credibility of the website (4-item Journal of the American Medical Association benchmark), accuracy (proportion of website treatment recommendations that were appropriate), and comprehensiveness of website treatment recommendations (proportion of guideline treatment recommendations that were appropriately covered by a website).

**Results:**

We included 79 websites from 6 English-speaking countries. In terms of credibility, 31% (25/79) of the websites clearly disclosed that they had been updated after the publication of the NICE guidelines. Only 43.28% (487/1125) website treatment recommendations were judged as accurate. Comprehensiveness of treatment recommendations correctly covered by websites was very low across all types of LBP. For acute LBP, an average of 28% (4/14) guideline recommendations were correctly covered by websites. Websites for radicular LBP were the least comprehensive, correctly covering an average of 16% (2.3/14) recommendations.

**Conclusions:**

Noncommercial freely accessible websites demonstrated low credibility standards, provided mostly inaccurate information, and lacked comprehensiveness across all types of LBP.

## Introduction

### Background

Low back pain (LBP) is the condition that accounts for the greatest burden of disability worldwide [1]. The global burden of LBP is expected to rise with an ageing population, leading to increased pressure on health systems [2]. The literature on LBP is vast, with a considerable amount of evidence on risk factors [3], prognosis [4], and effectiveness of treatment for LBP [5,6]. However, misconceptions in the general population and among health professionals [7] about LBP are very common [8,9]. A possible solution to reduce the inconsistency between beliefs of the general public and evidence-based information is to disseminate appropriate information through easily accessible means, such as the internet.

The internet has rapidly become an important source of health information [10]. In fact, there is evidence that more people seek health information first on the internet than with health care providers [11]. Recent data indicate that 78% [12] of Australians and 61% of people in the United Kingdom [13] used the internet to obtain health information in the previous 12 months [12,13]. People engaging with internet-based health information do so for several reasons, including making treatment decisions, supplementing information provided by a health professional, and self-managing a health condition [14]. The ready availability of internet-based health information may have both favorable and unfavorable consequences: although it empowers people to actively participate in their health care, the poor quality of information across many health conditions [15-17] calls into question its usefulness.

Government agencies and health care and professional organizations now sponsor websites containing health information for a broad range of health topics, including LBP. Websites hosted by those organizations are usually seen as more credible than other sources of internet-based information for people with LBP [18]. Previous studies examining the content of internet-based information about LBP have limitations, such as investigating limited samples and including websites with commercial purposes [19-21].

### Objectives

Our objective was to assess the credibility, accuracy, and comprehensiveness of treatment recommendations for LBP given by websites sponsored by sources typically recognized as trustworthy by the public.

## Methods

### Eligibility Criteria

We reported this review following guidance provided by the Preferred Reporting Items for Systematic Reviews and Meta-Analyses guidelines where possible [22]. We identified trustworthy websites to be those from government agencies, nonprofit nongovernmental organizations, hospitals, professional societies, universities, and consumer organizations. We sought websites from 6 major English-speaking countries: Australia, Canada, New Zealand, South Africa, the United Kingdom, and the United States. Websites had to present content about treatments for either acute, persistent, or radicular LBP. Websites that had links to other forms of content presentation, such as booklets, leaflets, or brochures, were also included. We excluded websites published in languages other than English, websites only containing information about aspects other than treatment of LBP (eg, anatomy of the lumbar spine), and those requiring any type of password or membership. We excluded websites that did not provide at least 1 clear recommendation for either acute, persistent, or radicular LBP.

### Search Strategy

We used Google to search for noncommercial freely accessible websites presenting LBP information. We conducted searches on 5 February and updated it on July 20, 2018. We used Google AdWords to identify the most popular terms on Google related to LBP worldwide. To increase search specificity, we conducted searches on each country’s dedicated Google website (Multimedia Appendix 1). Furthermore, 1 researcher (GF) performed the initial screening of websites using the Google Chrome Web browser. Before starting every new search, we cleared the browsing data. We screened the first 50 records from each search by looking at titles, snippets that followed each title, and URLs for each retrieved Web page. All links deemed relevant by the first reviewer were collated to a Microsoft Excel sheet and then screened for eligibility by 1 of the 3 independent reviewers (AT, CM, and GM), with all discrepancies resolved by discussion.

### Data Extraction and Coding

A reviewer (GF) extracted data into a spreadsheet and 1 of the 3 other reviewers (AT, GM, and MOK) cross-checked the data. We extracted information on characteristics of the website, such as the host, country, and type of LBP covered. Websites treatment recommendations were coded according to the recommendations from the 2016 National Institute for Health and Care Excellence (NICE) guidelines [5] and the 2017 American College of Physicians [6] guidelines for the management of LBP with or without sciatica. Furthermore, 3 authors (GF, AT, and CM) coded each guideline recommendation as having been (1) endorsed by at least 1 guideline; (2) dismissed by at least 1 guideline; and (3) subject to conflicting positions between the 2 guidelines. The accuracy of treatment recommendations given by websites was judged by concordance with the guideline recommendations and coded as follows:

- Appropriate endorsement: A recommendation given by a website to use a treatment that was endorsed by at least 1 guideline.
- Appropriate dismissal: A recommendation given by a website to avoid a treatment that was dismissed by at least 1 guideline.
- Inappropriate endorsement: A recommendation given by a website to use a treatment that was dismissed by at least 1 guideline.
- Inappropriate dismissal: A recommendation given by a website to avoid a treatment that was endorsed by at least 1 guideline.
- Endorsed: A recommendation given by a website to use a treatment that was not mentioned in either guideline.
- Dismissed: A recommendation given by a website to avoid a treatment that was not mentioned in either guideline.
- Unclear: A recommendation given by a website that was not clearly targeted to a specific LBP condition (eg, a recommendation to use skeletal muscle relaxants for LBP) or when a recommendation was vague in the description of the treatment (eg, *spinal injections* rather than *epidural corticosteroid injection* or *facet joint corticosteroid injection*).

### Outcomes

Our primary outcomes were credibility, accuracy, and comprehensiveness of each website.

Credibility: We used the 4-item Journal of the American Medical Association (JAMA) benchmark [23] to assess the credibility of each website. The JAMA benchmark addresses 4 elements: (1) currency of information, (2) declaration of authorship, (3) presentation of a list of references, and (4) disclosure of any conflict of interest, funding, or sponsorship. Each item was categorized as yes, no, or not reported. We considered a website to be up-to-date if its date of publication or last update had been subsequent to the publication date of the 2016 NICE guidelines [5]. We considered authorship to be declared when single or multiple authors were listed or when authorship was attributed to a working group or an entity.

Accuracy: We defined accuracy as the number and proportion of website recommendations that were judged clear and accurate. Accurate recommendations were those coded as being appropriate endorsements, appropriate dismissals, and treatments dismissed by the website and not listed in either guideline. Inaccurate recommendations were those coded as being inappropriate endorsements, inappropriate dismissals, and treatments endorsed by the website but not listed in either guideline.

Comprehensiveness: We defined comprehensiveness as the number and proportion of guideline recommendations that were appropriately covered by a website. The comprehensiveness of a website was given by the ratio between the sum of appropriate endorsements and dismissals and the total number of recommendations in the guidelines for each different type of LBP.

### Data Analysis

We presented data for acute, persistent, and radicular LBP separately. We used descriptive statistics to summarize credibility, accuracy, and comprehensiveness across the websites. Each item of the JAMA benchmark was presented individually. Data on accuracy were presented as the number and proportion of clear accurate recommendations, the number of clear accurate recommendations to use a treatment, and the number of clear accurate recommendations to avoid a treatment. Data on comprehensiveness were presented as the mean (SD) number of guideline recommendations correctly covered by websites and as the average proportion of website recommendations, as well as the mean (SD) number of guideline recommendations correctly covered by websites to use and to avoid a treatment.

## Results

### Selected Websites

**Figure 1:**
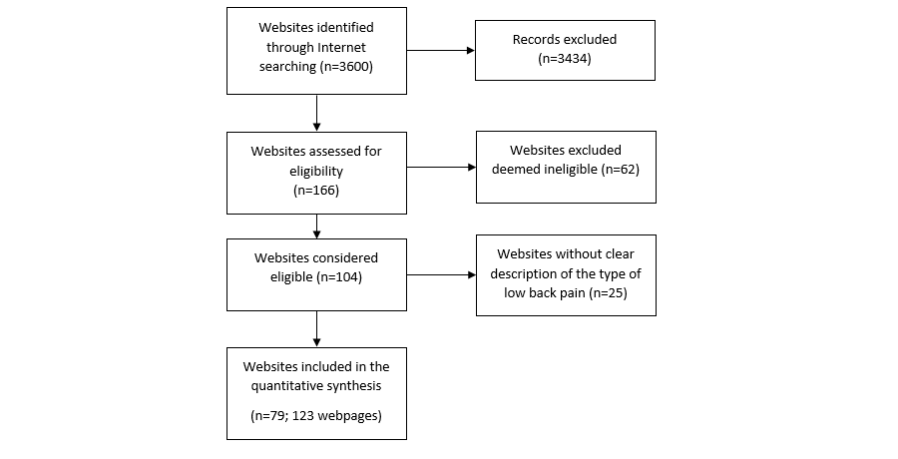
Study flow diagram.

We conducted 72 searches on Google, resulting in 3600 records to be screened. We excluded 3434 records by reading the titles on Google and retained 166 websites for eligibility assessment. Among these, 87 were ineligible. We, therefore, included 79 unique websites. As some websites had information for more than 1 type of LBP, a total of 123 Web pages were included in the final analysis (Figure 1).

### Characteristics of the Included Websites

Table 1 outlines the characteristics of the included websites. 53% (42/79) of the websites were from the United States, 24% (19/79) were from the United Kingdom, 17% (13/79) were from Australia, 4% (3/79) were from Canada, 1% (1/79) were from New Zealand, and 1% (1/79) were from South Africa. 43% (34/79) of websites were owned by hospitals, followed by websites from government agencies, which represented 25% (20/79) of websites. More information about the included websites is shown in Multimedia Appendix 2. Detailed information about the treatments covered by the websites is listed in Multimedia Appendix 3 to Multimedia Appendix 6.

### Credibility of Websites

A total of 43 websites (54.4%, 43/79) disclosed their creation date or last update, and only 25 (31%, 25/79) of these websites had been updated after the publication of the NICE guidelines. Only 18 (22%, 18/79) websites provided a declaration of authorship. Only 21 (26%, 21/79) websites presented a list of references. Disclosure of any potential conflict of interest, funding source, or any form of sponsorship was only provided by 5 (6%, 5/79) websites (Table 1). Details on the assessment of credibility are shown in Multimedia Appendix 2.

**Table 1:** Characteristics of websites and credibility data (N=79).

Descriptive and credibility variables		Statistics, n (%)
**Country**		
	Australia	13 (16.4)
	Canada	3 (3.8)
	New Zealand	1 (1.2)
	South Africa	1 (1.2)
	United Kingdom	19 (24.1)
	United States	42 (53.3)
**Type of website**		
	Consumer organization	2 (2.5)
	Government	20 (25.3)
	Hospital	34 (43.0)
	Nongovernmental organization	8 (10.1)
	Professional association or society	8 (10.1)
	University	7 (8.8)
**Type of low back pain^a^**		
	Acute	55 (69.6)
	Persistent	29 (36.7)
	Radicular	39 (49.3)
**Updated after 2016** **National Institute for Health and Care Excellence** **guidelines**		
	No	19 (24.1)
	Yes	25 (31.6)
	Not reported	35 (44.3)
**Declaration of authorship**		
	No	61 (77.2)
	Yes	18 (22.8)
**List of references**		
	No	58 (73.4)
	Yes	21 (26.6)
**Disclosure of any conflict of interest, funding, or sponsorship**		
	Yes	5 (6.3)
	Not reported	74 (93.7)

^a^The total is greater than 79 as some websites presented information for more than 1 type of low back pain (LBP).

**Table 2:** Accuracy of website treatment recommendations.

Condition	Number of recommendations	Number of unclear recommendations, n (%)	Number of accurate recommendations, n (%)	Number of clear accurate recommendations to use a treatment, n (%)	Number of clear accurate recommendations to avoid a treatment, n (%)
Acute LBP	452	98 (21.7)	228 (50.4)	187 (61.7)	41 (80.3)
Persistent LBP	402	105 (26.1)	154 (38.3)	116 (45.8)	38 (86.3)
Radicular LBP	271	28 (10.3)	105 (38.7)	86 (38.7)	17 (84.4)
Total	1125	231 (20.53)	487 (43.28)	389 (50.00)	96 (82.7)

**Table 3:** Comprehensiveness of website treatment recommendations. Recommendations that were conflicting between guidelines were not included in the assessment of comprehensiveness.

Condition	Number of recommendations in the guidelines	Guideline recommendations correctly covered by websites, mean (SD; percentage of total number of recommendations in guidelines)	Guideline recommendations to use a treatment correctly covered by websites, mean (SD; percentage of total number of recommendations in guidelines)	Guideline recommendations to avoid a treatment correctly covered by websites, mean (SD; percentage of total number of recommendations in guidelines)
Acute LBP^a^	14	4.0 (1.5; 28.6)	3.4 (1.3; 24.3)	0.6 (0.6; 4.3)
Persistent LBP	25	4.5 (2.8; 18.0)	4.0 (2.3; 16.0)	0.5 (1.1; 2.0)
Radicular LBP	14	2.3 (1.9; 16.4)	2.2 (1.9; 15.7)	0.1 (0.3; 0.7)

^a^LBP: low back pain.

### Accuracy of Website Recommendations

Data for accuracy are presented in Table 2. In total, websites provided 1125 recommendations, with 487 (43.3%) being accurate, 407 (36.2%) inaccurate, and 231 (20.5%) unclear. Websites provided many more recommendations to use rather than to avoid a treatment (778 vs 116). Acute LBP had the highest number of recommendations among all the types of LBP. The proportion of accurate recommendations for acute LBP (50.4%) was higher than the proportion of accurate recommendations for persistent LBP (38.3%) and radicular LBP (38.7%). Advice to stay active was the most common treatment recommendation given by websites for acute LBP, having been endorsed by 45 out of 55 (81%) websites. Moreover, 28 out of 55 (50%) websites inappropriately endorsed paracetamol for acute LBP, and 6 out of 29 (20%) websites inappropriately endorsed opioids for persistent LBP (Multimedia Appendices 3-5).

### Comprehensiveness of Website Recommendations

Details on the comprehensiveness of website recommendations are shown in Table 3. The proportion of guideline recommendations correctly covered by websites was higher for acute LBP (28.6%) compared with persistent LBP (18.0%) and radicular LBP (16.4%). Websites for radicular LBP were the least comprehensive, covering on average 2.3 (1.9) guideline recommendations. Across all types of LBP, the most comprehensive websites correctly covered only about 50% of the guideline recommendations—see Multimedia Appendix 6.

## Discussion

### Principal Findings

Treatment recommendations for LBP in websites from trustworthy sources failed to meet our benchmarks for credibility, provided a high proportion of inaccurate or unclear recommendations, and lacked comprehensiveness. In general, websites did not provide adequate resources for people to independently verify the truthfulness of the information provided. The accuracy of treatment recommendations was generally low across all different types of LBP. The lack of comprehensiveness across all websites was even more pronounced when covering guideline recommendations to avoid an ineffective treatment.

### Comparison With Previous Work

Previous research had surveyed the accuracy of information only for a specific type of LBP (eg, acute LBP) [20], had not distinguished between types of LBP when analyzing recommendations from guidelines [19,21], and examined a limited sample of websites [19-21,24], including commercial websites [19,21]. We presented results for acute, persistent, and radicular LBP separately, given that some treatments known to be effective for 1 type of LBP might be ineffective for another or evidence might be currently lacking or be of questionable quality. That is the case for skeletal muscle relaxants, where evidence supports their use for acute LBP but benefits for persistent and radicular LBP are still uncertain [25].

In contrast to previous studies [19-21,26], we surveyed only websites from sources recognized as trustworthy by patients with LBP [18], such as government agencies, health care organizations, and universities. On the basis of the findings of previous studies showing that commercial websites were mostly of poor quality [19,21], our a priori hypothesis was that noncommercial freely accessible websites would have more comprehensive and accurate information compared with commercial websites [19,21]. This was not the case for many guideline-endorsed treatments. For example, although advice to stay active was recommended for acute LBP by 81% (45/55) of the websites, only 41% (12/29) and 46% (18/39) of websites recommended it for radicular and persistent LBP, respectively. Our findings suggest that websites of trusted sources are failing not only at conveying the accurate message on the benefits of guideline-endorsed [5,6] and first-line treatment [27] recommendations but also at dismissing ineffective treatment options such as bed rest for acute LBP, for which evidence on its ineffectiveness has long been known [28].

The large number of recommendations that were inaccurate and unclear found in our review supports findings from previous studies that people cannot obtain appropriate information about LBP on the internet [18]. For example, more than half of all treatment recommendations given by websites in our review were either inaccurate or unclear, which risks misleading the public [29]. In addition, evidence from the general population suggests that seeking health-related information on the internet is associated with increased health care utilization [30-32]. The fact that people often seek health information on the internet for LBP combined with the large amount of inaccurate and unclear information contained in these websites can potentially be driving people to seek unnecessary or ineffective care. On the contrary, credible and accurate internet-based information may contribute to reducing health care utilization. A recent study has attributed a 12% decrease in overall health care utilization, including LBP, to the launching of a website containing guideline-endorsed information maintained by the Dutch College of General Practitioners [33]. If one of the goals of internet-based health information is to reduce unnecessary consultations in primary care [33], these sources of internet-based information about LBP must provide patients the necessary means to make informed decisions about their health care. For this to happen, improving the credibility standards, as well as providing accurate and comprehensive treatment recommendations is necessary. When recommending treatments for LBP, websites must rely extensively on the evidence provided by high-quality clinical practice guidelines such as the NICE guidelines [5] and American College of Physicians [6] guidelines.

### Limitations

The strengths of this study include using Google AdWords to develop search terms individuals actually use to find internet-based information about treatments for LBP. Searching the first 50 records of each search can also be considered a strength, as people searching for internet-based information usually do not look past the 10 or 20 first hits [34]. One potential limitation of our study was the use of Google as the sole Web browser to screen for websites. Nevertheless, our choice was based on the fact that Google is the most used search engine worldwide, Google has the best search validity (ie, returns links to websites that can be opened), and results from other engines usually highly overlap with those from Google [34]. Another limitation involves a very small number of inconsistencies (3 recommendations out of a total of 56, 5%) between the 2 guidelines. Nevertheless, that likely represents the uncertainty around the evidence for treatments for which there is currently no consensus, such as acupuncture [35]. We dealt with this limitation by choosing a more conservative approach and classified all endorsements and dismissals of treatments with inconsistent recommendations as being inappropriate.

### Conclusions

Websites from government agencies, consumer organizations, hospitals, nongovernmental organizations, professional associations, and universities demonstrated low credibility standards, provided mostly inaccurate information, and lacked comprehensiveness across all types of LBP. Our findings highlight the need for these organizations to reformulate their treatment recommendations to reflect current evidence in the management of LBP.

## Appendix

Multimedia Appendix 1 Search terms employed on Google.

Multimedia Appendix 2 Additional characteristics of the included websites.

Multimedia Appendix 3 Frequency (%) of websites endorsing or dismissing treatments mentioned in guidelines for acute low back pain (N=55).

Multimedia Appendix 4 Frequency (%) of websites endorsing or dismissing treatments mentioned in guidelines for persistent low back pain (N=28).

Multimedia Appendix 5 Frequency (%) of websites endorsing or dismissing treatments mentioned in guidelines for radicular low back pain (N=39).

Multimedia Appendix 6 Accuracy and comprehensiveness of website recommendations.

## Abbreviations

JAMA: Journal of the American Medical Association
LBP: low back pain
NICE: National Institute for Health and Care Excellence
