# Supplementary material for: Credibility, Accuracy, and Comprehensiveness of Internet-Based Information About Low Back Pain: A Systematic Review
Source: J Med Internet Res. 2019 May 7;21(5):e13357. doi: 10.2196/13357 (PMC6529212; doi:10.2196/13357)
Supplement: Multimedia Appendix 1 [file jmir_v21i5e13357_app1.pdf]

Multimedia Appendix 1. Search terms employed on Google

**Low back pain**	***Sciatica***
Australia (www.google.com.au)	
**Government:** low back pain Australia gov<br>**NGOs:** low back pain Australia org<br>**Consumer organisations**: low back pain Australia consumer reports<br>**Hospitals:** low back pain Australia hospital<br>**Universities**: low back pain Australia university<br>**Professional associations/societies**: low back pain Australia professional societies	**Government:** low back pain Australia gov<br>**NGOs:** low back pain Australia org<br>**Consumer organisations**: low back pain Australia consumer reports<br>**Hospitals:** low back pain Australia hospital<br>**Universities**: low back pain Australia university<br>**Professional associations/societies**: low back pain Australia professional societies
Canada (www.google.ca)	
**Government:** low back pain Canada gov<br>**NGOs:** low back pain Canada org<br>**Consumer organisations:** low back pain Canada consumer<br>**Hospitals:** low back pain Canada hospital services<br>**Universities:** low back pain Canada university<br>**Professional associations/societies:** low back pain Canada association society	**Government:** low back pain Canada gov<br>**NGOs:** low back pain Canada org<br>**Consumer organisations:** low back pain Canada consumer<br>**Hospitals:** low back pain Canada hospital services<br>**Universities:** low back pain Canada university<br>**Professional associations/societies:** low back pain Canada association society
New Zealand (www.google.co.nz)	
**Government:** low back pain New Zealand govt<br>**NGOs:** low back pain New Zealand org<br>**Consumer organisations:** low back pain New Zealand consumer<br>**Hospitals:** low back pain New Zealand hospital<br>**Universities:** low back pain university ac.nz<br>**Professional associations/societies:** low back pain New Zealand Society	**Government:** low back pain New Zealand govt<br>**NGOs:** low back pain New Zealand org<br>**Consumer organisations:** low back pain New Zealand consumer<br>**Hospitals:** low back pain New Zealand hospital<br>**Universities:** low back pain university ac.nz<br>**Professional associations/societies:** low back pain New Zealand Society
South Africa (www.google.co.za)	
**Government**: low back pain South Africa gov.za	**Government**: sciatica pain South Africa gov.za

**NGOs:** low back pain South Africa org.za	**NGOs:** sciatica South Africa org.za
**Consumer organisations:** low back pain Africa consumers	**Consumer organisations:** sciatica Africa consumers
**Hospitals:** low back pain hospital Africa .za	**Hospitals:** sciatica hospital Africa .za
**Universities:** low back pain South Africa university	**Universities:** sciatica South Africa university
**Professional associations/societies:** low back pain South Africa professional association	**Professional associations/societies:** sciatica South Africa professional association
United Kingdom (www.google.co.uk)	
**Government:** low back pain gov.uk	**Government:** low back pain gov.uk
**NGOs:** low back pain uk org	**NGOs:** low back pain uk org
**Consumer organisations:** low back pain uk consumer reports	**Consumer organisations:** low back pain uk consumer reports
**Hospitals:** low back pain uk hospital	**Hospitals:** low back pain uk hospital
**Universities:** low back pain uk university	**Universities:** low back pain uk university
**Professional associations/societies:** low back pain uk association society	**Professional associations/societies:** low back pain uk association society
United States (www.google.com)	
**Government:** low back pain .gov usa	**Government:** low back pain .gov usa
**NGOs:** low back pain org usa	**NGOs:** low back pain org usa
**Consumer organisations:** low back pain consumer usa	**Consumer organisations:** low back pain consumer usa
**Hospitals:** low back pain hospital usa	**Hospitals:** low back pain hospital usa
**Universities:** low back pain U.S university health services	**Universities:** low back pain U.S university health services
**Professional associations/societies:** low back pain U.S association society	**Professional associations/societies:** low back pain U.S association society
