# Supplementary material for: Credibility, Accuracy, and Comprehensiveness of Internet-Based Information About Low Back Pain: A Systematic Review
Source: J Med Internet Res. 2019 May 7;21(5):e13357. doi: 10.2196/13357 (PMC6529212; doi:10.2196/13357)
Supplement: Multimedia Appendix 2 [file jmir_v21i5e13357_app2.pdf]

Multimedia Appendix 2. Additional characteristics of the included websites (N = 79)

Website	Type of website	Condition	Presentation of content	JAMA benchmark			
				Updated after NICE Guidelines	Declaration of authorship	List of references	Disclosure of any conflict of interest, funding or sponsorship
**Australia**							
Arthritis Australia	Non-governmental organisation	Acute, persistent	Booklet	No	Yes	No	Yes
Australian Pain Management Association	Non-governmental organisation	Radicular	Website	NR	No	No	NR
Australian Physiotherapy Association	Professional society/association	Acute, persistent, radicular	Website	Yes	Yes	No	NR
Better Health	Government	Acute, persistent, radicular	Website	Yes	No	Yes	NR
Choice	Consumer organisation	Acute, persistent	Website	Yes	Yes	No	NR
Choosing Wisely Australia	Professional society/association	Acute	Website	No	No	No	NR
Chronic Pain Australia	Non-governmental organisation	Acute	Fact sheet Website	NR	No	No	NR
Health Direct	Government	Acute, radicular	Website	No	No	Yes	NR
Health Queensland	Government	Acute	Fact sheet	NR	No	No	NR
Healthy Western Australia	Government	Acute, persistent	Brochure	No	No	No	NR
Musculoskeletal Australia	Consumer organisation	Acute, persistent, radicular	Website	Yes	No	No	NR
South Australia Government	Government	Acute, radicular	Fact sheet	No	No	Yes	NR
Tasmania Government	Government	Acute	Booklet	No	Yes	No	NR
**Canada**							
Choosing Wisely Canada	Professional society/association	Acute	Website	NR	No	No	NR
HealthLinkBC (British Columbia Government)	Government	Radicular	Website	Yes	Yes	No	NR
Institute of Health Economics (IHE)	Government	Acute, persistent	Booklet	No	No	No	NR
**New Zealand**							
Choosing wisely New Zealand	Professional society/association	Acute	Website	NR	No	Yes	NR

**South Africa**							
Department of Health	Government	Acute	Website	Yes	No	No	NR
**The United Kingdom**							
Arthritis Research UK	Non-Governmental organisation	Acute, persistent	Booklet	No	No	Yes	NR
Barts Health	Hospital	Acute	Booklet	Yes	No	Yes	NR
British Association of Spine Surgeons (BASS)	Professional society/association	Acute	Website	No	No	No	NR
East Lancashire Hospital	Hospital	Acute, Persistent	Booklet	Yes	No	No	NR
Focus on Disability	Non-Governmental organisation	Radicular	Website	NR	No	No	NR
Guy's and Saint Thomas'	Government	Radicular	Leaflet	Yes	No	No	NR
Hampshire County Council	Government	Acute	Booklet	NR	No	No	NR
Health and Safety Executive	Government	Acute	Website	NR	No	No	NR
Healthshare (Oxfordshire)	Non-Governmental organisation	Acute, Persistent, radicular	Website	NR	No	No	NR
Ipswich Hospital	Hospital	Acute	Booklet	Yes	No	Yes	NR
NHS Choices	Government	Acute, radicular	Website	Yes	No	No	NR
NHS Direct Wales	Government	Radicular	Website	Yes	No	No	NR
NiDirect	Government	Acute	Website	Yes	No	No	NR
Oxford University Hospitals	Hospital	Acute	Booklet	No	No	No	NR
Poole Hospital	Hospital	Acute, persistent, radicular	Booklet	Yes	Yes	Yes	NR
Queen Victoria Hospital	Hospital	Persistent	Booklet	No	No	No	NR
Royal Berkshire ED patient sheet	Hospital	Acute, persistent	Booklet	Yes	No	Yes	NR
The University of Nottingham Health Service	University	Acute	Website	NR	Yes	No	NR
York Teaching Hospital	Hospital	Acute	Website	NR	No	No	NR
**The United States**							
Agency for Healthcare Research and Quality (AHRQ)	Government	Acute, persistent	Website	Yes	Yes	Yes	NR
American Academy of Family Physicians	Professional society/association	Acute	Website	Yes	No	No	NR
American Chronic Pain Association	Non-governmental organisation	Acute, persistent, radicular	Booklet	No	Yes	No	NR

American Physical Therapy Association (APTA)	Professional society/association	Acute, radicular	Website	No	No	Yes	NR
Beaumont Hospital	Hospital	Radicular	Website	NR	No	No	NR
Cedars-Sinai	Hospital	Acute, persistent, radicular	Website	NR	No	No	NR
Cleveland Clinic	Hospital	Acute, persistent, radicular	Website	Yes	No	Yes	NR
Columbia Spine Hospital	Hospital	Radicular	Website	NR	No	No	NR
El Camino Hospital	Hospital	Acute, radicular	Website	NR	No	No	NR
Emory Healthcare	Hospital	Radicular	Website	NR	No	No	NR
Hospital for Special Surgery (HSS)	Hospital	Acute, persistent, radicular	Website	No	Yes	Yes	Yes
International Association of Firefighters	Non-governmental organisation	Acute	Website	NR	No	No	NR
Johns Hopkins University	Hospital	Acute, persistent	Website	NR	No	No	NR
Massachusetts General Hospital	Hospital	Unclear	Website	NR	No	No	NR
Mayo Clinic	Hospital	Acute, persistent, radicular	Website	NR	No	No	NR
MedlinePlus	Government	Acute, persistent	Website	No	Yes	Yes	NR
Mount Sinai Hospital	Hospital	Radicular	Website	NR	No	No	NR
National Center for Complementary and Integrative Health (NCCIH)	Government	Persistent	Website	No	Yes	Yes	NR
National Institute of Arthritis and Musculoskeletal and Skin Diseases (NIAMS)	Government	Acute, persistent	Website	No	No	No	NR
National Institute of Neurological Disorders and Stroke (NINDS)	Government	Acute, persistent	Website	Yes	No	No	NR
New England Baptist Hospital	Hospital	Acute, radicular	Website	NR	No	No	NR
North America Spine Surgeons Society (NASS)	Professional society/association	Acute	Booklet	No	Yes	No	NR
Oregon Health & Sciencex'	University	Radicular	Website	NR	No	No	NR
Penn Medicine	Hospital	Acute, radicular	Website	Yes	Yes	Yes	NR
Rush Hospital	Hospital	Radicular	Website	NR	No	NO	NR
SSM Health	Hospital	Radicular	Website	NR	No	No	NR

St Lukes	Hospital	Acute, persistent	Website	Yes	Yes	Yes	Yes
Univerity of Minnesota medical Center	Hospital	Radicular	Website	NR	No	No	NR
University of Berkeley	University	Acute	Website	NR	No	No	NR
University of California	University	Acute	Website	NR	No	No	NR
University of Florida Health	Hospital	Persistent	Website	Yes	Yes	Yes	NR
University of Kansas	Hospital	Persistent	Website	NR	No	No	NR
University of Miami hospital and clinics	University	Radicular	Website	NR	No	No	NR
University of Michigan	University	Radicular	Website	Yes	Yes	Yes	Yes
University of Notre Dame	University	Acute	Leaflet	No	No	No	NR
University of Rochester Medical Center	Hospital	Radicular	Website	NR	No	No	NR
University of Stanford health Care	Hospital	Radicular	Website	NR	No	No	NR
University of Utah Health	Hospital	Acute, persistent, radicular	Website	NR	No	No	NR
University of Vermont medical center	Hospital	Acute, persistent, radicular	Website	Yes	No	No	NR
University of Virginia health system	Hospital	Radicular	Website	NR	No	No	NR
University of Winsconsin health	Hospital	Acute, persistent, radicular	Website	Yes	Yes	No	Yes
Winchester Hospital	Hospital	Radicular	Website	NR	No	Yes	NR

NR, not reported;
