# Supplementary material for: Credibility, Accuracy, and Comprehensiveness of Internet-Based Information About Low Back Pain: A Systematic Review
Source: J Med Internet Res. 2019 May 7;21(5):e13357. doi: 10.2196/13357 (PMC6529212; doi:10.2196/13357)
Supplement: Multimedia Appendix 3 [file jmir_v21i4e13357_app3.docx]

**Supplementary file 3**. Frequency (%) of websites endorsing or dismissing treatments mentioned in guidelines for acute low back pain (n= 55)

| Treatment | Appropriate endorsement | Inappropriate endorsement | Appropriate dismissal | Inappropriate dismissal | Unclear recommendation | Omissions |
| --- | --- | --- | --- | --- | --- | --- |
| Treatments endorsed by at least one guideline | | | | | | |
| Advice to stay active | 45 (81.8) | - | - | 2 (3.6) | 1 (1.8) | 7 (12.7) |
| Education | 6 (10.9) | - | - | 0 (0) | 2 (3.6) | 47 (85.4) |
| Exercise | 34 (61.8) | - | - | 1 (1.8) | 6 (10.9) | 14 (25.4) |
| Heat | 37 (67.2) | - | - | 0 (0) | 6 (10.9) | 12 (21.8) |
| Massage | 8 (14.5) | - | - | 0 (0) | 2 (3.6) | 45 (81.8) |
| Muscle relaxants | 9 (16.3) | - | - | 2 (3.6) | 1 (1.8) | 43 (78.1) |
| Non-steroidal anti-inflammatory | 35 (63.6) | - | - | 4 (7.3) | 2 (3.6) | 14 (25.4) |
| Psychologically-informed physical therapy | 0 (0) | - | - | 0 (0) | 0 (0) | 55 (100) |
| Spinal manipulative therapy | 12 (21.8) | - | - | 0 (0) | 4 (7.3) | 39 (70.9) |
| Weak opioids* | 1 (1.8) | 9 (16.3) | 1 (1.8) | 0 (0) | 5 (9.1) | 39 (70.9) |
| Treatments dismissed by at least one guideline | | | | | | |
| Bed rest | - | 4 (7.2) | 2 (3.6) | - | 3 (5.5) | 46 (83.6) |
| Paracetamol | - | 28 (50.9) | 26 (47.3) | - | 0 (0) | 1 (1.8) |
| Strong opioids | - | 6 (10.9) | 0 (0) | - | 5 (9.1) | 44 (80) |
| Systemic corticosteroids | - | 1 (1.8) | 0 (0) | - | 1 (1.8) | 53 (96.3) |
| Conflicting recommendations |  |  |  |  |  |  |
| Acupuncture | - | 4 (7.3) | - |  | 5 (9.1) | 46 (83.6) |

*Some values may not add up to 100% due to rounding*

**Prescribing weak opioids was considered appropriate only when (1) prescription of other medicines was not possible (e.g. any counter indication) (2) as last resort when other medicines failed at improving pain*
