# Supplementary material for: Credibility, Accuracy, and Comprehensiveness of Internet-Based Information About Low Back Pain: A Systematic Review
Source: J Med Internet Res. 2019 May 7;21(5):e13357. doi: 10.2196/13357 (PMC6529212; doi:10.2196/13357)
Supplement: Multimedia Appendix 4 [file jmir_v21i5e13357_app4.pdf]

Multimedia Appendix 4. Frequency (%) of websites endorsing or dismissing treatments mentioned in guidelines for persistent low back pain (N=29)

Treatment	Appropriate endorsement	Inappropriate endorsement	Appropriate dismissal	Inappropriate dismissal	Unclear recommendation	Omissions
Treatments endorsed by at least one guideline						
Advice to stay active	12 (41.3)	-	-	0 (0)	1 (3.4)	16 (55.2)
Behavioural (operant) therapy	2 (6.9)	-	-	0 (0)	0 (0)	27 (93.1)
Cognitive behavioural therapy	6 (20.7)	-	-	0 (0)	0 (0)	23 (79.3)
Education	8 (27.6)	-	-	0 (0)	1 (3.4)	20 (68.9)
Exercise	23 (79.3)	-	-	1 (3.4)	2 (6.9)	3 (10.3)
Low level laser therapy	0 (0)	-	-	1 (3.4)	1 (3.4)	27 (93.1)
Massage	12 (41.3)	-	-	0 (0)	4 (13.8)	13 (44.8)
Mindfulness	1 (3.4)	-	-	1 (3.4)	0 (0)	27 (93.1)
Multidisciplinary treatment	10 (34.4)	-	-	0 (0)	0 (0)	19 (65.5)
Non-steroidal anti-inflammatory	14 (48.3)	-	-	0 (0)	3 (10.3)	12 (41.3)
Progressive relaxation	5 (17.2)	-	-	0 (0)	0 (0)	24 (82.8)
Radiofrequency denervation	2 (6.9)	-	-	0 (0)	0 (0)	27 (93.1)
Spinal manipulative therapy	11 (37.9)	-	-	1 (3.4)	6 (20.7)	11 (37.9)
Tai Chi	2 (6.9)	-	-	0 (0)	0 (0)	27 (93.1)
Yoga	8 (27.6)	-	-	0 (0)	3 (10.3)	18 (62.1)
Treatments dismissed by at least one guideline						
Antidepressants (tricyclic)	-	4 (13.8)	3 (10.3)	-	6 (20.7)	16 (55.2)
Benzodiazepines	-	1 (3.4)	0 (0)	-	1 (3.4)	27 (93.1)
Epidural corticosteroid injection	-	3 (10.3)	0 (0)	-	8 (27.6)	18 (62.1)
Facet joint corticosteroid injection	-	2 (6.9)	0 (0)	-	9 (31.0)	18 (62.1)
Kinesio taping	-	0 (0)	0 (0)	-	0 (0)	29 (100)
Opioids (any)	-	9 (31.0)	2 (6.9)	-	3 (10.3)	15 (51.7)
Paracetamol	-	6 (20.7)	2 (6.9)	-	4 (13.8)	17 (58.6)
Spinal fusion	-	4 (13.8)	3 (10.3)	-	6 (20.7)	16 (55.2)
TENS	-	2 (6.9)	2 (6.9)	-	5 (17.2)	20 (68.9)
Ultrasound	-	1 (3.4)	2 (6.9)	-	1 (3.4)	25 (86.2)
Conflicting recommendations						
Acupuncture	-	17 (58.6)		-	2 (6.9)	10 (34.4)

Some values may not add up to 100% due to rounding
