# Supplementary material for: Credibility, Accuracy, and Comprehensiveness of Internet-Based Information About Low Back Pain: A Systematic Review
Source: J Med Internet Res. 2019 May 7;21(5):e13357. doi: 10.2196/13357 (PMC6529212; doi:10.2196/13357)
Supplement: Multimedia Appendix 5 [file jmir_v21i5e13357_app5.pdf]

Supplementary file 5. Frequency (%) of websites endorsing or dismissing treatments mentioned in guidelines for radicular low back pain (n= 39)

Treatment	Appropriate endorsement	Inappropriate endorsement	Appropriate dismissal	Inappropriate dismissal	Unclear recommendation	Omissions
Treatments endorsed by at least one guideline						
Advice to stay active	18 (46.1)	-	-	0 (0)	1 (2.5)	20 (51.2)
Antidepressant (Duloxetine)	1 (2.5)	-	-	0 (0)	0 (0)	38 (97.4)
Exercise	24 (61.5)	-	-	0 (0)	0 (0)	15 (38.4)
Epidural corticosteroid injection	13 (33.3)	-	-	1 (2.5)	9 (23.1)	16 (41.0)
Gabapentin	6 (15.4)	-	-	0 (0)	1 (2.5)	32 (82.0)
Multidisciplinary treatment	0 (0)	-	-	0 (0)	0 (0)	39 (100)
Opioid (only as acute rescue)	0 (0)	-	-	0 (0)	4 (10.2)	35 (89.7)
Pregabalin	5 (12.8)	-	-	0 (0)	1 (2.5)	33 (84.6)
Spinal manipulative therapy	4 (10.2)	-	-	0 (0)	1 (2.5)	34 (87.1)
Spinal decompression	15 (38.4)	-	-	1 (2.5)	2 (5.1)	21 (53.8)
Treatments dismissed by at least one guideline						
Acupuncture	-	6 (15.4)	0 (0)	-	0 (0)	33 (84.6)
Benzodiazepines (Diazepam)	-	2 (5.1)	0 (0)	-	0 (0)	37 (94.8)
Systemic corticosteroids	-	2 (5.1)	1 (2.5)	-	0 (0)	36 (92.3)
Traction	-	3 (7.7)	1 (2.5)	-	0 (0)	35 (89.7)
Conflicting recommendations						
Antidepressant (Amitriptyline)	-	4 (10.2)	0 (0)	-	0 (0)	35 (89.7)

Some values may not add up to 100% due to rounding

**Prescribing opioids was considered appropriate only as acute rescue – the smallest dose for the shortest period*
