# Supplementary material for: Credibility, Accuracy, and Comprehensiveness of Internet-Based Information About Low Back Pain: A Systematic Review
Source: J Med Internet Res. 2019 May 7;21(5):e13357. doi: 10.2196/13357 (PMC6529212; doi:10.2196/13357)
Supplement: Multimedia Appendix 6 [file jmir_v21i5e13357_app6.pdf]

Multimedia Appendix 6. Accuracy and comprehensiveness of website recommendations

Website	Total no of recommendations given by the website	AE	IE	AD	ID	UNC	END	DIS	No of clear accurate recommendations	No of recommendations from guidelines correctly covered by the website (%)
Acute LBP										
Arthritis Australia	19	4	3	1	0	9	1	1	6	5 (35.7%)
Australia Physiotherapy Association	6	4	0	1	1	0	0	0	5	5 35.7%)
Better Health (Victoria Government)	1	1	0	0	0	0	0	0	1	1 (7.1%)
Choice	18	4	2	2	0	9	1	0	6	6 (42.9%)
Choosing Wisely Australia	7	2	1	1	0	0	3	0	3	3 (21.4%)
Chronic Pain Australia	6	2	1	2	0	0	0	1	5	4 (28.6%)
Health Direct	13	5	2	0	1	3	1	1	6	5 (35.7%)
Health Queensland (Queensland government)	6	3	0	0	2	0	1	0	3	3 (21.4%)
Healthy WA (Western Australia Government)	6	3	1	1	0	0	1	0	4	4 (28.6%)
Musculoskeletal Australia	8	4	0	1	0	1	2	0	5	5 (35.7%)
South Australia Government	3	2	0	1	0	0	0	0	3	3 (21.4%)
Tasmania Government	7	3	1	1	0	0	2	0	4	4 (28.6%)
Acute_Institute of Health Economics (IHE)	31	6	3	2	0	14	1	5	6	6 (42.9%)
Choosing Wisely Canada	9	5	0	1	0	0	3	0	13	8 (57.1%)
Choosing Wisely New Zealand	5	2	1	0	0	0	2	0	2	2 (14.3%)
Department of Health - South Africa	12	5	1	0	1	0	3	2	7	5 (35.7%)
Arthritis Research UK	18	0	0	0	0	18	0	0	0	0 (0.0%)
Barts Health - NHS Trust	7	5	1	0	0	0	1	0	5	5 (35.7%)
British Association of Spine Surgeons (BASS)	5	2	2	1	0	0	0	0	3	3 (21.4%)
East Lancashire Hospital	7	4	1	1	0	0	1	0	5	5 (35.7%)
Hampshire County Council	4	2	0	1	0	0	1	0	3	3 (21.4%)
Health and Safety Executive	5	4	0	0	0	0	1	0	4	4 (28.6%)
Healthshare (Oxfordshire)	3	2	0	1	0	0	0	0	3	3 (21.4%)
Ipswich Hospital	4	3	0	0	0	0	1	0	3	3 (21.4%)
NHS Choices	8	6	0	0	0	0	2	0	6	6 (42.9%)
NiDirect	6	4	1	1	0	0	0	0	5	5 (35.7%)
Oxford University Hospitals	3	2	0	0	0	0	1	0	2	2 (14.3%)
Poole Hospital	12	5	4	1	0	0	2	0	6	6 (42.9%)
Royal Berkeshire Hospital	6	4	1	0	0	0	1	0	4	4 (28.6%)
The University of Nottingham Health Service	7	4	2	0	0	0	1	0	4	4 (28.6%)
York Teaching Hospital	4	3	1	0	0	0	0	0	3	3 (21.4%)
Agency for Healthcare Research and Quality (AHRQ)	5	4	0	1	0	0	0	0	5	5 (35.7%)

American Academy of Family Physicians	7	5	1	0	0	0	1	0	5	5	(35.7%)
American Chronic Pain Association	13	3	1	0	0	9	0	0	3	3	(21.4%)
American Physical Therapy Association (APTA)	8	5	0	1	0	0	2	0	6	6	(42.9%)
Cedars-Sinai	6	4	1	0	0	0	1	0	4	4	(28.6%)
Cleveland Clinic	5	3	1	0	0	0	1	0	3	3	(21.4%)
El Camino Hospital	5	1	0	0	0	0	4	0	1	1	(7.1%)
Hospital for Special Surgery (HSS)	12	4	2	1	0	0	5	0	5	5	(35.7%)
International association of firefighters	8	5	1	1	0	0	1	0	6	6	(42.9%)
Johns Hopkins Medicine	4	2	1	0	1	0	0	0	2	2	(14.3%)
Mayo Clinic	17	3	0	1	0	12	1	0	4	4	(28.6%)
MedlinePLus	6	4	1	1	0	0	0	0	5	5	(35.7%)
National Institute of Arthritis and Musculoskeletal and Skin Diseases (NIAMS)	5	2	1	0	1	0	0	1	3	2	(14.3%)
National Institute of Neurological Disorders and Stroke (NINDS)	13	4	3	1	0	3	2	0	5	5	(35.7%)
New England Baptist Hospital	8	4	2	0	0	0	2	0	4	4	(28.6%)
North American Spine Society (NASS)	10	5	1	1	0	2	1	0	6	6	(42.9%)
Penn Medicine	6	2	0	1	1	0	2	0	3	3	(21.4%)
St Lukes	12	3	0	0	0	8	1	0	3	3	(21.4%)
University of Berkeley	4	2	0	0	1	0	1	0	2	2	(14.3%)
University of Davis - California	7	5	1	0	0	0	1	0	5	5	(35.7%)
University of Notre Dame	7	3	2	0	1	0	1	0	3	3	(21.4%)
University of Utah	14	4	3	1	0	4	2	0	5	5	(35.7%)
University of Vermont medical center	6	2	1	0	0	2	1	0	2	2	(28.6%)
University of Winsconsin	8	3	0	0	0	4	1	0	3	3	(28.4%)
Persistent LBP											
Arthritis Australia	21	4	1	1	0	12	1	2	7	5	(20.0%)
Australia Physiotherapy Association	8	3	0	4	0	0	0	1	8	7	(28.0%)
Better Health (Victoria Government)	10	5	0	0	0	0	4	1	6	5	(20.0%)
Choice	18	4	1	2	0	9	2	0	6	6	(24.0%)
Healthy WA (Western Australia Government)	4	4	0	0	0	0	0	0	4	4	(16.0%)
Musculoskeletal Australia	2	1	0	0	0	0	1	0	1	1	(4.0%)
Institute of Health Economics (IHE)	38	9	5	4	3	11	3	3	16	13	(52.0%)
Arthritis Research UK	21	1	0	0	0	17	0	3	4	1	(4.0%)
East Lancashire	6	3	1	0	0	0	1	1	4	3	(12.0%)
Healthshare (Oxfordshire)	16	4	2	0	0	5	4	1	5	4	(16.0%)
Poole Hospital	2	2	0	0	0	0	0	0	2	2	(8.0%)
Queen Victoria Hospital	7	2	0	0	0	0	4	1	3	2	(8.0%)
Royal Berkeshire Hospital	15	5	5	0	0	3	1	1	6	5	(20.0%)

Agency for Healthcare Research and Quality (AHRQ)	13	8	2	1	0	0	2	0	9	9	(36.0%)
American Chronic Pain Association	15	6	1	0	0	8	0	0	6	6	(24.0%)
Cedars-Sinai	14	4	1	0	0	0	9	0	4	4	(16.0%)
Cleveland Clinic	7	3	3	0	0	0	1	0	3	3	(12.0%)
Hospital for special surgery (HSS)	6	3	2	0	0	0	1	0	3	3	(12.0%)
Johns Hopkins Medicine	15	3	1	0	0	2	9	0	3	3	(12.0%)
Mayo Clinic	13	0	1	0	0	12	0	0	0	0	(0.0%)
MedlinePLus	15	5	3	0	0	1	6	0	5	5	(20.0%)
National Center for Complementary and Integrative Health (NCCIH)	7	4	1	0	0	1	1	0	4	4	(16.0%)
National Institute of Arthritis and Musculoskeletal and Skin Diseases (NIAMS)	20	4	3	0	0	4	9	0	4	4	(16.0%)
National Institute of Neurological Disorders and Stroke (NINDS)	24	5	3	1	2	3	8	2	8	6	(24.0%)
St Lukes	30	9	3	0	0	11	4	3	12	9	(36.0%)
University of Florida Health	16	5	4	0	0	0	6	1	6	5	(20.0%)
University of Kansas	10	4	2	0	0	3	1	0	4	4	(16.0%)
University of Utah	24	6	4	1	1	2	8	2	9	7	(28.0%)
University of Winsconsin	5	0	0	0	0	1	2	2	2	0	(0.0%)
Radicular LBP											
Australia Pain Management Association	3	2	0	0	0	1	0	0	2	2	(14.3%)
Australian Physiotherapy Association	8	2	0	2	1	1	1	1	5	4	(28.6%)
Better Health (Victoria Government)	13	5	2	0	0	0	5	1	6	5	(35.7%)
Health Direct	6	1	0	0	0	2	3	0	1	1	(7.1%)
South Australia Government	3	2	0	0	0	0	0	1	3	2	(14.3%)
Health Link - British Columbia	9	2	0	0	0	3	4	0	2	2	(14.3%)
Arthritis Research UK	6	4	0	0	0	0	2	0	4	4	(28.6%)
Focus on disabiilty	16	6	2	0	0	1	7	0	6	6	(42.9%)
Guy's and Saint Thomas'	1	1	0	0	0	0	0	0	1	1	(7.1%)
Healthshare (Oxfordshire)	8	4	0	0	0	0	3	1	5	4	(28.6%)
NHS Choices	10	3	0	0	0	0	5	2	5	3	(21.4%)
NHS Direct - Wales	20	8	3	0	0	0	8	1	9	8	(57.1%)
Poole Hospital	6	4	1	0	0	0	1	0	4	4	(28.6%)
American Chronic Pain Association	4	4	0	0	0	0	0	0	4	4	(28.6%)
American Physical Therapy Association (APTA)	7	2	1	0	0	0	3	1	3	2	(14.3%)
Beaumont Hospital	4	1	1	0	0	0	2	0	1	1	(7.1%)
Cedars-Sinai	5	2	1	0	0	0	2	0	2	2	(14.3%)
Cleveland Clinic	10	3	1	0	0	0	6	0	3	3	(21.4%)

Columbia Spine Hospital	5	2	0	0	0	1	2	0	2	2	(14.3%)
El Camino Hospital	3	0	1	0	0	1	1	0	0	0	(0.0%)
Emory Healthcare	4	1	0	0	0	0	3	0	1	1	(7.1%)
Hospital for Special Surgery	5	1	0	0	1	0	3	0	1	1	(7.1%)
Mayo Clinic	13	6	2	0	0	1	4	0	6	6	(42.9%)
Mount Sinal Hospital	4	2	0	0	0	0	2	0	2	2	(14.3%)
New England Baptist Hospital	7	3	1	0	0	0	3	0	3	3	(21.4%)
Oregon Health & Science University	6	0	0	0	0	0	6	0	0	0	(0.0%)
Penn University Hospital	9	1	0	0	0	4	3	1	2	1	(7.1%)
Rush University Medical Center	5	3	0	0	0	0	2	0	3	3	(21.4%)
SSM Health	4	0	1	0	0	0	3	0	0	0	(0.0%)
University of Miami Hospital	4	1	1	0	0	0	2	0	1	1	(7.1%)
University of Michigan	8	2	1	0	0	1	4	0	2	2	(14.3%)
University of Minnesota medical center	6	0	2	0	0	2	2	0	0	0	(0.0%)
University of Rochester Medical Centre	4	1	1	0	0	0	2	0	1	1	(7.1%)
University of Stanford Health	7	1	1	0	0	3	2	0	1	1	(7.1%)
University of Utah	9	3	1	0	0	4	1	0	3	3	(21.4%)
University of Vermont medical center	2	0	1	0	0	0	1	0	0	0	(0.0%)
University of Virginia	9	2	1	0	0	0	6	0	2	2	(14.3%)
University of Winsconsin	6	1	1	0	0	0	4	0	1	1	(7.1%)
Winchester Hospital	12	0	1	0	0	3	0	8	8	0	(0.0%)

LBP, low back pain; AE, appropriate endorsement, AD, appropriate dismissal; IE, inappropriate endorsement, ID, inappropriate dismissal; UNC, unclear; END, endorsed; DIS, dismissed
